# Arabidopsis Regenerating Protoplast: A Powerful Model System for Combining the Proteomics of Cell Wall Proteins and the Visualization of Cell Wall Dynamics

**DOI:** 10.3390/proteomes4040034

**Published:** 2016-11-17

**Authors:** Ryusuke Yokoyama, Hiroaki Kuki, Takeshi Kuroha, Kazuhiko Nishitani

**Affiliations:** Laboratory of Plant Cell Wall Biology, Graduate School of Life Sciences, Tohoku University, Sendai 890-8578, Japan; ryokoyama@m.tohoku.ac.jp (R.Y.); kuki@biology.tohoku.ac.jp (H.K.); tkuroha@m.tohoku.ac.jp (T.K.)

**Keywords:** protoplast, cell wall, sub-proteomics, imaging technique

## Abstract

The development of a range of sub-proteomic approaches to the plant cell wall has identified many of the cell wall proteins. However, it remains difficult to elucidate the precise biological role of each protein and the cell wall dynamics driven by their actions. The plant protoplast provides an excellent means not only for characterizing cell wall proteins, but also for visualizing the dynamics of cell wall regeneration, during which cell wall proteins are secreted. It therefore offers a unique opportunity to investigate the de novo construction process of the cell wall. This review deals with sub-proteomic approaches to the plant cell wall through the use of protoplasts, a methodology that will provide the basis for further exploration of cell wall proteins and cell wall dynamics.

## 1. Introduction

Plant cell walls are complex and dynamic cellular structures that play a critical role not only in determining cell shape, but also in developmental processes, intercellular communication and defensive responses [1,2,3,4]. One of the main features of the plant cell wall is a framework composed of cellulose microfibrils embedded in a matrix of polysaccharides, such as hemicelluloses and pectins [5,6,7,8,9]. However, approaches to visualizing the structural dynamics of this framework remain underdeveloped, despite its essential roles in determining the physical properties and regulating the biological functions of the cell wall. Thus, the development of a new, reliable method enabling the visualization of cell wall dynamics is a high priority of research aimed at a more refined understanding of cell wall formation, both in terms of the synthesis and integration of new polysaccharides and the assembly of the polysaccharide network.

The assembly and rearrangement of the cell wall structure are mainly achieved *in muro* via the actions of extracellular cell wall proteins [10]. The abundance and diversity of cell wall proteins, which account for approximately 10% of the cell wall mass, contribute to the structural and functional diversity of the cell wall [11]. Each of the complete plant genome sequences reported to date is estimated to contain at least several thousand genes encoding putative extracellular proteins [12]. Only a limited number of these extracellular proteins has so far been characterized for function, particularly regarding cell wall dynamics [9,13], and thus, a full picture of how cell wall dynamics result from the concerted action of such proteins is not yet attainable.

Protoplasts isolated enzymatically from the tissues and cultured cells of plants are capable of forming new cell walls and therefore offer a unique opportunity to study various steps of cell wall construction *de novo*. We previously produced protoplasts from suspension-cultured cells of *Arabidopsis* and, using histochemical staining techniques and electron microscopy, observed cell wall dynamics at the cell surface during cell wall regeneration [14]. Furthermore, using two-dimensional polyacrylamide gel electrophoresis (2D PAGE) and matrix-assisted laser desorption ionization-time-of-flight/mass spectrometry (MALDI-TOF/MS), we successfully identified approximately three hundred extracellular proteins derived from regenerated protoplasts and suspension-cultured cells. In this review, we will introduce these techniques, summarize some applications involved in recent developments and, finally, discuss some open problems.

## 2. Preparation of Protoplasts and Cell Wall Regeneration from Protoplasts

**Figure 1:**
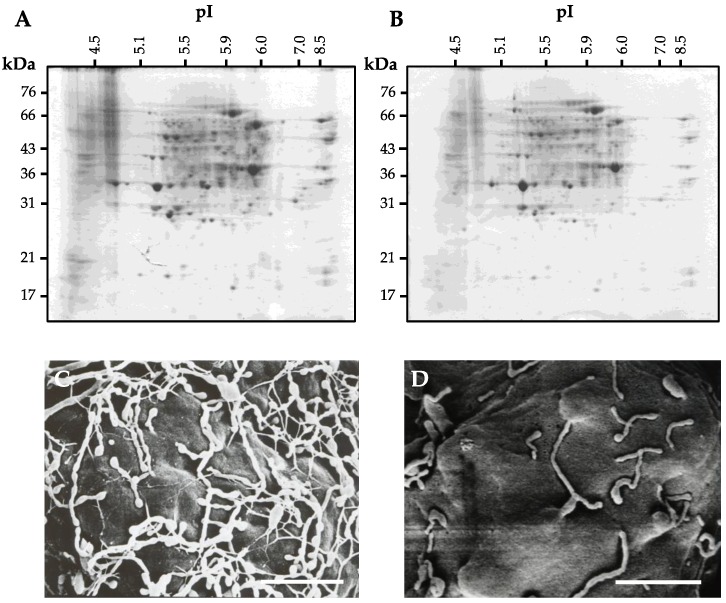
Comparative expression pa/erns of cell wall proteins and scanning electron microscope images of cell walls in the protoplasts regenerated for 3 h in the absence (**A**,**C**) or presence (**B**,**D**) of 1 μM DCB. 2-D PAGE analysis of cell wall proteins from the 3-h cell-wall regenerated protoplasts (**A**) and the 3-h cell-wall regenerated protoplast treated with 1 μM DCB (**B**). The cell wall proteins were prepared using the nonBdisruptive extraction with 1 M KCl, and separated on a 3–10 linear pI gradient in the first dimension and visualized using Coomassie staining. Scanning electron microscope images of the protoplasts regenerated in the absence of DCB was adapted from Kwon et al. [14]. Scanning electron microscopic analysis was performed according to a similar procedure described by Kwon et al. [14]. Bar = 1.5 µm.

The suspension-cultured Alex cell line of *Arabidopsis*, which was established by Mathur et al. [15], has proven highly useful for analyzing large quantities of relatively uniform *Arabidopsis* cells. In a previous study, we prepared protoplasts from suspension-cultured Alex cells; the protocol is available at the website (https://www.plantcellwall.jp/protocol/pdf/protocol_11.pdf) [16]. In this system, the protoplasts produce a new cell wall in a short time and also show a relatively high level of synchrony of cell wall regeneration. The merits of using protoplasts include the ability to treat cells directly with chemical reagents or cell wall enzymes, such as glycoside hydrolases; for example, protoplasts treated with 2,6-dichlorobenzonitrile (DCB), an inhibitor of cellulose synthase [17], immediately ceased synthesis of cellulose, and this was followed by changes in the expression pattern of cell wall proteins (Figure 1).

The application of reverse genetics to the protoplast-based cell wall regeneration system also provides an attractive approach for characterizing cell wall proteins. It is not easy, however, to establish a suspension culture cell line with specific genes knocked out. We therefore recently developed an improved procedure for the regeneration of cell walls in protoplasts derived from mesophyll cells of *Arabidopsis* rosette leaves [18] to take advantage of T-DNA insertion lines, which are currently available for more than 20,000 genes in *Arabidopsis* (http://signal.salk.edu/index.html) [19]. Using this procedure, a large amount of protoplasts can easily be prepared from fully-expanded rosette leaves of three- to five-week-old *Arabidopsis* plants. Although the protoplasts derived from leaf mesophyll cells show slightly lower levels of synchrony of cell wall regeneration than cultured cells, the efficiency of cell wall regeneration is high, and more than 90% of protoplasts regenerate cell walls. In addition, transcriptomic analysis using microarray technology confirmed that most of the genes identified by proteomic analysis as encoding cell wall proteins in regenerating protoplasts derived from suspension-cultured Alex cells were also expressed in protoplasts derived from mesophyll (Table S1). This improved protoplast system is thus amenable to reverse genetics.

## 3. Visualization of Cell Wall Dynamics in Regenerating Protoplast 

The understanding of the structural aspects of the plant cell wall has been inspired and guided by biochemical analysis. In our study, simple measurements of the sugar composition of cell walls from the protoplasts suggested gradual changes in the proportion of cell wall polysaccharides, such as pectin, with progress in regeneration (Tables S2 and S3). However, it is notoriously difficult to elucidate cell wall dynamics convincingly using only biochemical analysis (Tables S2 and S3). To gain insight into the dynamic aspects of cell wall formation, we used histochemical methods to visualize β-glucans deposited on the surface of protoplasts. Cell wall-regenerating protoplasts were stained with either Calcofluor White M2R or aniline blue, which preferentially stain cellulose and β-1,3 glucan, respectively. Both epifluorescent images were observed under a fluorescence microscope equipped with a UV fluorescence filter set (excitation filter: 350 nm; barrier filter: 430 nm). The deposition of glucans, as determined by fluorescent staining, appeared within 1 h of cell wall regeneration, and the extent of deposition increased and spread across the surface over 3 h of regeneration [14].

Recently, we examined in more detail the sequence of deposition of cellulose microfibrils in regenerating protoplasts using a confocal laser scanning microscope [18]. The protoplasts showed a random orientation of cellulose microfibrils in the early stages, followed by partially unidirectional reorientation of cellulose microfibrils. This implies that ordered cellulose deposition may be responsible for the early polarization of protoplasts and the oriented expansion of regenerated protoplasts.

Visualization of cellulose microfibrils using fluorescence staining with Calcofluor White has been performed in many plant species [20,21,22]. Although this procedure is simple and robust, Calcofluor White has the disadvantage of specificity for cellulose microfibril and toxic properties that might be injurious to the live cells. Recently, fluorescence staining with Pontamine Fast Scarlet 4 BS (S4B) combined with spinning disk confocal microscopy has been used successfully to observe cellulose patterning [23]. This fluorescent dye binds cellulose with high specificity and has the potential to permit the staining of living cells [24]. The S4B staining protocol may be more valuable for real-time imaging of cellulose microfibrils of living cells. These studies strongly support the hypothesis that oriented deposition of cellulose microfibrils determines the direction of cell elongation [25]. However, the dynamics of the matrix polysaccharides associated with cellulose microfibrils during the early stage of the cell wall formation remain elusive, as do the subsequent dynamics of the reorientation of the cellulose microfibrils themselves.

In addition to staining with fluorescent compounds, immunofluorescence analysis using monoclonal antibodies is a broadly useful technique for visualizing cell wall polysaccharides [26,27,28]. The use of a disk-shaped plasma membrane sheet, prepared by the lysis of cell wall-regenerating protoplasts in a low osmotic medium, has greatly facilitated immunofluorescence analysis of cell wall polysaccharides [29,30].

**Figure 2:**
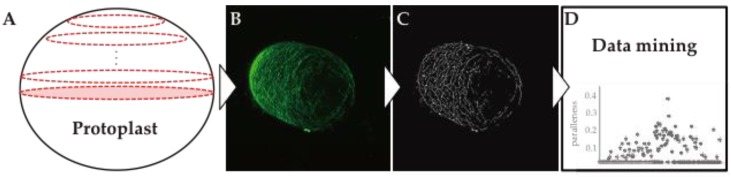
Schematic outline of the quantitative image analysis used in this study. (**A**) Histochemical or immunohistochemical staining of the cell wall components, and acquisition of serial optical image sections using confocal laser scanning microscopy; (**B**) Stacks of the serial optical sections; (**C**) Image processing and optimization; (**D**) Quantitative image analysis for total length, parallelness and straightening of the cell wall components.

These tools have helped identify cell wall polysaccharides in regenerating protoplasts and provided new insights into the organization of polysaccharides. Further progress in integrating the dynamics of each component into a complete picture of cell wall dynamics, however, may require new approaches. Quantitative imaging is one of the most useful techniques for evaluating the configuration and dynamics of the entire cell wall (Figure 2). Visualizing the dynamics of actin microfilaments and cortical microtubules, including their elongation, severing, buckling and straightening, is important in building up a complete picture of the cell wall [31,32]. The dynamic images acquired will provide a rich source of information, leading to a precise understanding of how the spatiotemporal organization of cell wall polysaccharides is governed by an ensemble of cell wall proteins.

## 4. Preparation of Cell Wall Proteins

### 4.1. Conventional Extraction Procedure for Cell Wall Proteins

The cell wall proteins loosely bound to the cell wall can be easily extracted with a series of solvents from the suspension cell culture medium and from intact cells [33,34]. In addition, the use of this non-destructive extraction procedure without disrupting cellular integrity can avoid contamination from cytoplasmic proteins. Robertson et al. [35] initially reported the systematic extraction of the cell wall proteins with a series of solvents, including aqueous solutions of 200 mM calcium chloride, 50 mM 1,2-cyclohexanediaminetetraacetic acid (CDTA), 2 mM dithiothreitol (DTT), 1 M sodium chloride and 200 mM borate buffer. This sequential washing approach, with some modifications, has been extensively used to obtain the cell wall proteins [36,37].

Bayer et al. [38] also summarized critical paths in plant cell wall proteomics: (1) sampling of plant tissues comprised of mixtures of cell types with differing cell wall characteristics; (2) preparation in pure form of the cell wall; (3) isolation and characterization of many proteins tightly associated with the cell wall; (4) the resolving ability of 2-DE for separation of some hydrophobic and many basic proteins. In view of the above problems associated with the sampling and preparation of the cell wall proteins, the use of uniform protoplasts, which can undergo synchronized regeneration of the cell wall, has proven to be useful in obtaining highly pure protein samples at the specific stage of the cell wall formation.

### 4.2. Extraction Procedure for Cell Wall Proteins from Protoplast

Comparison of the extraction efficiency and specificity of cell wall proteins from cell wall-regenerating protoplasts between various extraction solutions indicates that the highly efficient extraction of cell wall proteins can only be achieved using an extraction solution containing 1 M KCl. Glucose-6-phosphate dehydrogenase (G6PDH), a marker enzyme for contamination by cytoplasmic proteins, is used to determine the occurrence and extent of the leakage of cytosolic proteins from the protoplasts during the extraction processes [39]. In typical extraction experiments, the specific G6PDH activity in 1 M KCl extracts from protoplasts is less than 1% of that in cytosolic extracts.

A similar extraction method of cell wall proteins has also been used to study plant defense responses using suspension-cultured cells, to easily obtain the cell wall proteins secreted by the application of various stimuli [40,41]. In these studies, cell wall proteins could be extracted from the suspension-cultured cells, following the application of fungal elicitors, with calcium chloride. Thus, the use of protoplasts and cell cultures has been useful to easily obtain cell wall proteins. However, since the plasma membrane may easily be ruptured in these procedures, we should validate whether cytosolic proteins leak out of the protoplasts and the suspension-cultured cells during protein extraction processes. Today, we can use a number of different types of cytosolic markers [42] and discriminate between intracellular and extracellular proteins through bio-informatics analyses [43,44].

### 4.3. Problem of the Extraction Procedure for Cell Wall Proteins

Although the non-destructive solvent extraction approach is very useful for obtaining cell wall proteins without contamination, it may not be a satisfactory method for the comprehensive identification of all cell wall proteins. Some proteins are tightly linked to the cell wall architecture, even in the early stages of cell wall regeneration in protoplasts, and others are anchored to the plasma membrane. To overcome these problems, intrinsic to proteins tightly bound to the cell wall, modified series of solvents have been successfully used for non-destructive extraction [33,45,46]. Another problem intrinsic to the non-destructive extraction of proteins from protoplasts at early stages of cell wall regeneration is contamination with cytosolic proteins due to the rupture of the plasma membrane during sample handling [33,47].

### 4.4. Cell Wall Protein Purification Techniques

Some disruptive purification approaches coupled with a specific recovery procedure for cell wall proteins may produce a more comprehensive sample of isolated cell wall proteins. For example, *N*-glycosylation is a common post-translational modification for proteins trafficking through the secretory pathway, and therefore, most cell wall proteins are thought to be glycosylated in the apoplast [48,49,50]. For this reason, lectin-based affinity chromatography appears a promising method for obtaining cell wall proteins from a crude protein extract. More than one hundred glycoproteins were isolated from mature stems of *Arabidopsis thaliana* by affinity chromatography using Concanavalin A Sepharose [51]. Fluid-phase partitioning methods have also been widely used to isolate cell wall-related proteins, such as glycosylphosphatidyl inositol-anchored proteins (GAPs) and membrane proteins [52,53,54,55]. Finally, one of the most widely-available approaches is the extraction of proteins from a cell wall fraction, which has been separated from cell homogenates [56]. This approach has been further developed to enable the extraction of cell wall proteins with differing degrees of affinity to the cell wall fraction [57,58]. In *Saccharomyces cerevisiae* and other fungi, the extraction of proteins tightly bound to the cell wall has been achieved by enzymatic digestion of the isolated cell wall fraction [59,60,61]. Such an enzymatic digestion can also be applied to the extraction of cell wall proteins from plant cell homogenates.

### 4.5. Improved Extraction Procedure for Cell Wall Protein from Protoplasts

In general, a complete cell wall proteome cannot be obtained using a single protocol. Thus, to increase the efficiency of the extraction of cell wall proteins from protoplasts, it may be necessary to use a combination of non-destructive and disruptive procedures. One challenge to our sub-proteomic approach is the application of enzymatic digestion techniques to the non-disruptive method of extraction. By using enzymes capable of degrading cell wall polysaccharides, we sought to isolate proteins that were tightly associated with the cell wall from intact protoplasts. Although additional cell wall proteins could be isolated by this procedure, a decrease in cellular integrity caused by enzyme treatment may cause contamination with intracellular proteins. We need to further investigate the optimum enzymatic digestion technique to avoid contamination.

## 5. Protein Separation and Mass Spectrometry for Identification

In our study, cell wall proteins were separated by 2D PAGE using immobilized isoelectric point gradient strips (pI 3–10 and pI 6–11) for the first dimension separation and 12% SDS-PAGE for the second separation. After staining with coomassie brilliant blue (CBB) to visualize the protein spots, we mapped each protein in the 2D gels using Image Master 2D-Elite software (Amersham Pharmacia Biotech, Buckinghamshire, UK, v4.01). Individual protein spots were picked and in-gel digested with trypsin, and protein sequences and genes encoding the proteins were identified using MALDI-TOF/MS analysis coupled with a database search using the Mascot sequence database search program (Matrix Science, Boston, MA, USA). As a result, 108 protein spots were identified from protoplasts regenerated for 1 h and 116 spots from those regenerated for 3 h, while 55 protein spots were identified from the suspension-cultured Alex cells [14]. The identified proteins were classified according to the predicted families of the cell-wall related proteins [62] and other families, including proteases, esterases, kinases and oxidoreductases.

In our experiment, some spots in the 2D gels resulted in frustrated attempts to identify the corresponding protein by the peptide mass mapping using MALDI-TOF/MS. There are useful complements to this standard procedure. An alternative to the peptide mass mapping using MALDI-TOF/MS is *de novo* sequencing by electrospray ionization tandem mass spectrometry (ESI)-MS/MS. ESI-MS/MS yields amino acid sequences of the separated peptides, allowing more definitive identification of the protein [47]. The additional level of separation offered by liquid chromatography is thought to have several advantages for the increased resolving of the digested peptides. Additionally, recently, a method for the pre-fractionation of proteins previous to LC, such as a filter-aided sample preparation (FASP), may facilitate accurate mass spectrometers [63]. Thus, liquid chromatography coupled with tandem mass spectrometry (LC-MS/MS) may become one of the essential tools in our proteomic analysis.

There are also some problems particular to the cell wall proteins in the cell wall proteomics approach. For example, arabinogalactan proteins (AGPs), in which up to 90% of the polysaccharides are linked to the protein by *O*-glycosylation, are not revealed by staining with CBB and are instead recognized by the β-glucosyl Yariv reagent [64]. Identification of proteoglycans with high *O*-glycosylation, such as AGPs, requires deglycosylation prior to digestion with trypsin; anhydrous hydrogen fluoride treatment can be used for chemical deglycosylation of AGPs [65]. Additionally, recent progress in pre-fractionation of proteins may also be useful for identifying some particular cell wall proteins. Multidimensional Protein Identification Technology (MudPIT) is a good example of peptide pre-fractionation methods and can be used to resolve peptide mixtures prior to LC-MS analysis [66]. 2D PAGE analysis followed by peptide mass mapping using MALDI-TOF/MS is the simplest approach for the comparative identification of proteins in proteomic analysis. Its combination with additional techniques for specific applications may increase the coverage and accuracy of cell wall proteomics.

The *Arabidopsis* cell wall proteins, which are identified by means of proteomics, can be characterized by The Arabidopsis Information Resource (TAIR; http://arabidopsis.org). Although the biological functions of many of these proteins identified a decade ago have not yet been determined [14], recent progress in *Arabidopsis* genes has begun to shed light on the functions of these proteins. A good example is the characterization of AtMSR2 (At1g51630), which was annotated as an “unknown” gene in 2005 [14] and was recently demonstrated to be involved in the biosynthesis of galactomannan [67]. Table 1 summarizes the genes encoding carbohydrate-related enzymes whose functions have been re-characterized based on the recent studies.

In the TAIR database, however, there are still many cell wall proteins, which have no experimentally-defined function. Although it is difficult to elucidate the precise enzymatic function and biological role of each cell wall protein *in muro*, *in silico* analysis may generate a more comprehensive catalog of the cell wall proteins. The ATTED-II database (http://atted.jp/) of genes co-expressed in *Arabidopsis* is used to identify potential interactions among the list of the target genes [78,79]. If there are well-characterized proteins in a network of the target genes, knowledge of the well-characterized proteins can be useful for the subsequent assignment of function to the other unknown proteins in an interaction network. Furthermore, an empirically-determined protein-protein interaction database will facilitate the functional characterization of the unknown proteins [80]. In our study, we filtered the associated genes, which were identified in the regenerating protoplasts, through the ATTED-II co-expression database and constructed a large network (Figure S2). The enriched Gene Ontology (GO) terms for the large network included ‘cell wall’ (GO:0005618, *p*-value = 1.3 × 10^−17^) and ‘carbohydrate metabolic process’ (GO: 0005975, *p*-value = 2.2 × 10^−17^). In the network, we could find the well-characterized genes involved in the metabolism of cell wall polysaccharides, such as xyloglucan.

## 6. Xyloglucan Metabolism in Regenerating Protoplasts

Xyloglucan is the major hemicellulose for forming a basic framework of the cell wall. We focused on the elucidation of linking the identified proteins involved in xyloglucan metabolism during early stages of cell wall regeneration. *AtXTH11* (At3g48580) has been identified as a member of the endotransglucosylase/hydrolase (XTH) family of proteins [81,82,83], which can mediate splitting and/or reconnection of xyloglucan crosslinks [84,85,86,87,88]. This enzyme was present in both cell wall-regenerating protoplasts and suspension culture cells, implying that the metabolism of xyloglucan molecules is involved in cell walls.

*AtXYL1* (At1g68560) and *BGAL10* (At5g63810) have been confirmed as chiefly responsible for xyloglucan α-xylosidase activity and xyloglucan β-galactosidase activity, respectively [74,76,89]. Several potentially phosphorylated protein spots on the 2D polyacrylamide gel were identified as AtXYL1 by staining with Pro-Q Diamond dye (Molecular Probes, Eugene, OR, USA), and thus, this protein was predicted to undergo extensive post-translational modification [14]. Recent studies showed that dephosphorylation by purple acid phosphatase (PAP) decreased the activity levels of xyloglucan α-xylosidase, subsequently increasing the levels of xyloglucan oligosaccharides [90]. We confirmed that PAP1 (At1g13750) appears in both protoplasts regenerated for 1 h and in suspension culture cells, but not in protoplasts regenerated for 3 h. It should be noted that xyloglucan oligosaccharides, which are potential substrates of xyloglucan α-xylosidase and XTH, are incorporated into the cell plate and involved in the construction of the new cell wall, thereby promoting cell division, as well as cell expansion [91,92]. In addition, XTH itself is found not only in expanding tissues [93], but also in the cell plate during the construction of the new cell wall [94,95]. All of these observations suggest that the metabolism of xyloglucan molecules contributes considerably to the early construction of the cell wall in regenerating protoplasts.

The presence of xyloglucan molecules in the cell plate has been reported in earlier studies [96,97,98]. On the other hand, our analysis of the immune-disk recently showed that xyloglucan molecules stick to cellulose microfibrils at a very early stage of cell wall regeneration in protoplasts (Figure S1). Further imaging analysis, combined with reverse genetics, will provide new insights into the biological function of xyloglucan-related enzymes and, particularly, how xyloglucan molecules are modified and how the xyloglucan/cellulose networks are assembled during the early stages of cell wall construction.

## 7. Concluding Remarks 

A wide range of cell wall proteomic approaches have been developed, leading to our current understanding of plant cell walls [99,100,101]. These approaches have been complemented by the availability of an advanced database (WallProtDB; http://www.polebio.lrsv.ups-tlse.fr/WallProtDB/) [102]. *Arabidopsis* cell wall proteins, identified by means of proteomics, can also be characterized using The *Arabidopsis* Information Resource (TAIR; http://arabidopsis.org). In addition, the ATTED-II database (http://atted.jp/) of co-expressed genes in *Arabidopsis* may be used to identify potential interactions between genes [94,95]. We filtered the genes encoding proteins for a putative secretory signal peptide, which were identified in the regenerating protoplasts, through the ATTED-II co-expression database and constructed a large network (Figure S2). The enriched GO terms for this network included “cell wall” and “carbohydrate metabolic process”. Although a combination of proteomic approaches and *in silico* analysis is generating a more comprehensive catalog of cell wall proteins, it remains difficult to elucidate the precise enzymatic function and biological role of each protein *in muro*.

**Figure 3:**
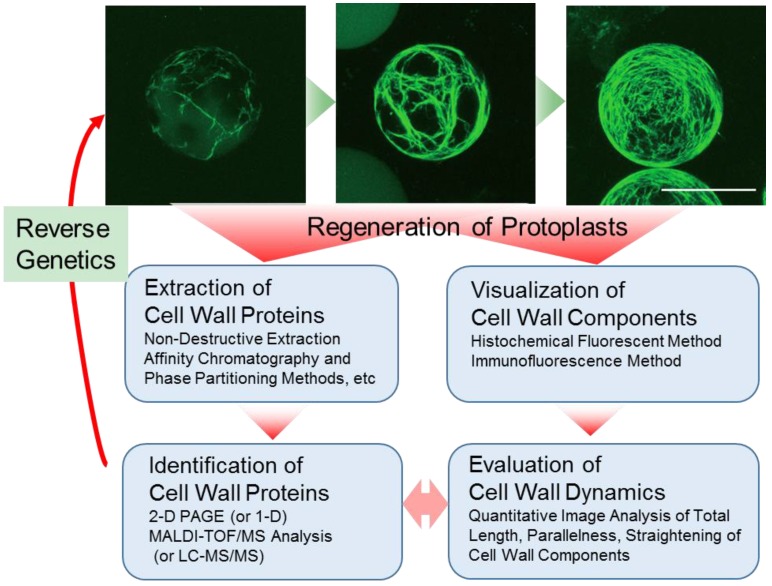
Strategies of cell wall sub-proteomic approaches of the cell wall, combined with the quantitative imaging technique and reverse genetics, using *Arabidopsis* mesophyll protoplast. Two approaches result in the comprehensive identification of the cell wall proteins and visualization of cell wall dynamics in the same stages of the protoplasts, leading to the prediction of the relationship between cell wall dynamics and the actions of the cell wall proteins. Furthermore, by use of the protoplasts derived from the T-DNA-insertion line, in which a certain gene of interest is disrupted, quantitative imaging analysis of cell wall regeneration would facilitate the identification of cell wall-related genes responsible for cell wall dynamics and help assign the precise biological role to the gene product.

Protoplasts are a useful model system for the comprehensive identification of cell wall proteins and for visualizing cell wall dynamics. Further progress in integrating reverse genetics into this approach will greatly facilitate the assignment of precise biological roles to these proteins and a better understanding of cell wall dynamics (Figure 3).

Finally, the recent extensive availability of complete genome sequences, and subsequent integrated omics analyses, will allow this protoplast system to be applied to other plant species. The diversity of cell wall composition and structure between plant species is well known [103,104,105,106]; as, for example, cell walls of commelinoid monocots, such as rice (*Oryza sativa*), contain relatively little xyloglucan and instead include a large amount of glucuronoarabinoxylan [2,107,108]. We have already developed an imaging technique for rice protoplasts and clarified some differences in the pattern of cellulose deposition (unpublished). Application of the protoplast system will provide new insights into the significant differences in cell wall dynamics between plant species.

## Figures and Tables

**Table 1:** Carbohydrate-related proteins in the regenerating protoplasts and suspension-cultured cells.

Family *	AGI	Description **	Protein Name	1 h	2 h	Native	References
GH1	At1g66280	β-Glucosidase	Bglu22	+	+		
GH1	At2g44450	β-Glucosidase	Bglu15			+	
GH1	At3g09260	β-Glucosidase	Bglu23/PYK10		+		Ogasawara et al., 2009 [68]
GH3	At5g20950	β-Xylosidase		+	+	+	
GH9	At1g71380	β-1,4-Glucanase	CEL3			+	Lewis et al., 2013 [69]
GH16	At3g48580	Xyloglucan	XTH11	+	+	+	Yokoyama et al., 2010 [70]
		endotransglucosylase/hydrolase					
GH18	At5g24090	Chitinase	CHIA			+	Takenaka et al., 2009 [71]
GH19	At2g43610	Chitinase		+			
GH27	At5g08380	α-Galactosidase	AGAL1	+	+	+	
GH28	At5g41870	Polygalacturonase				+	Cao, 2012 [72]
GH28	At5g06860	Polygalacturonase	PGIP1			+	Ferrari et al., 2012 [73]
GH31	At1g68560	α-Xylosidase	AXY3/XYL1	+	+	+	Günl and Pauly, 2011 [74]
GH32	At3g13790	β-Fructosidase	CWI	+	+	+	Mazola et al., 2015 [75]
GH35	At5g63810	β-Galactosidase	BGAL10	+	+	+	Sampedro et al., 2012 [76]
GH38	At3g26720	α-Mannosidase		+	+	+	
EXP	At3g45970	Expansin	EXPL1			+	

* Gene family names are defined in the Carbohydrate-Active Enzymes (CAZy) database [77], except for expansin; ** Descriptions are based on the descriptions of definition lines provided for characterized members of the family, as well as gene annotation records. The proteins identified in the 1-h regenerated protoplasts, the 3-h regenerated protoplasts and suspension cells are represented by + in the 1 h, 3 h and Native columns, respectively.

## Supplementary Materials

The following are available online at www.mdpi.com/2227-7382/4/4/34/s1: Table S1: Comparison of cell wall proteins of protoplasts derived from suspension-cultured cells and mesophyll cells in *Arabidopsis*; Table S2: Composition of the supernatant collected after every extraction step of the cell wall preparation; Table S3: Proportion of monosaccharides in the pectin fraction; Figure S1: Localization of xyloglucan molecules and β-glucan on plasma membrane sheets of the regenerating protoplasts; Figure S2: The co-expression network of the genes identified in the regenerating protoplasts.
