# Supplementary material for: Arabidopsis Regenerating Protoplast: A Powerful Model System for Combining the Proteomics of Cell Wall Proteins and the Visualization of Cell Wall Dynamics
Source: Proteomes. 2016 Nov 17;4(4):34. doi: 10.3390/proteomes4040034 (PMC5260967; doi:10.3390/proteomes4040034)
Supplement: Supplementary file 1 [file proteomes-04-00034-s001.pdf]

# Supplementary Materials: Arabidopsis Regenerating Protoplast—A Powerful Model System for Combining Proteomics of Cell Wall Proteins and Visualization of Cell Wall Dynamics

**Ryusuke Yokoyama, Hiroaki Kuki, Takeshi Kuroha and Kazuhiko Nishitani**

**Table S1.** Comparison of cell wall proteins of protoplasts derived from suspension-cultured cells and mesophyll cells in *Arabidopsis*.

| Protein Name * | AGI | Microarray Analysis ** | | | |
|---|---|---|---|---|---|
| | | 0 h | 6 h | 12 h | 24 h |
| Aspartyl Protease | At1g03230 | + | + | + | + |
| Malate dehydrogenase | At1g04410 | + | + | + | + |
| Glyceraldehyde-3-phosphate dehydrogenase | At1g13440 | + | + | + | + |
| Purple acid phosphatase | At1g13750 | + | + | + | + |
| Thioredoxin | At1g21350 | + | + | + | + |
| FAD-linked oxidoreductase | At1g30730 | + | + | + | + |
| Legume lectin | At1g53070 | | + | + | + |
| Myrosinase-associated protein | At1g54000 | | + | | + |
| Myrosinase-associated protein | At1g54010 | + | | + | + |
| β-Hexosaminidase | At1g65590 | + | + | + | + |
| β-Glucosidase | At1g66280 | | | | |
| Xyloglucan α-Xylosidase | At1g68560 | + | + | + | + |
| Strictosidine synthase | At1g74000 | + | + | + | + |
| Curculin-like lectin | At1g78850 | + | + | + | + |
| Serine protease | At2g04160 | + | + | + | + |
| Chitinase | At2g43610 | + | + | + | + |
| 6-Phosphogluconate dehydrogenase | At3g02360 | + | + | + | + |
| Glyceraldehyde-3-phosphate dehydrogenase | At3g04120 | + | + | + | + |
| β-Glucosidase | At3g09260 | | | | |
| Cell-wall invertase | At3g13790 | + | + | + | + |
| GDSL-motif esterase | At3g14220 | + | + | + | + |
| Legume lectin | At3g15356 | + | + | + | + |
| Legume lectin | At3g16530 | + | + | + | + |
| α-Mannosidase | At3g26720 | + | + | + | + |
| GDSL-motif lipase/hydrolase | At3g27950 | | | | |
| Xyloglucan endotransglucosylase/hydrolase | At3g48580 | + | + | + | + |
| 20S proteasome beta subunit | At4g14800 | + | + | + | + |
| Phosphorylase | At4g24340 | + | + | + | + |
| Aldose 1-epimerase | At4g25900 | + | + | + | + |
| Monodehydroascorbate reductase | At5g03630 | + | + | + | + |
| α-Galactosidase | At5g08380 | + | + | + | + |
| Leucine-rich repeat protein | At5g12940 | + | | | |
| Nucleoside hydrolase | At5g18860 | + | + | | |
| β-Xylosidase | At5g20950 | + | + | + | + |
| TRAF-like family protein | At5g26260 | + | + | + | + |
| TRAF-like family protein | At5g26280 | | | | |
| FAD-linked oxidoreductase | At5g44380 | + | + | + | + |
| Xyloglucanβ-Galactosidase | At5g63810 | + | + | + | + |

* The genes encoding the proteins with putative secretory signal peptide identified in the regenerated protoplasts from the suspension-cultured Alex cells. Descriptions are based on the descriptions of definition lines provided for characterized members of the family, as well as gene annotation records. ** The data based on microarray analysis of the mesophyll protoplast. The genes encoding the apoplastic proteins identified in the 0-h, 6-h, 12-h and 24-h regenerated protoplasts are represented by + in the 6 h, 12 h and 24 h columns, respectively.

**Microarray Analysis**

Total RNA was isolated from the mesophyll protoplasts regenerated for 0 h, 6 h, 12 h and 24 h, using an RNeasy Plant Mini Kit (Qiagen, CA, USA). Each RNA was labeled and amplified using a Low Input Quick Amplification/Labeling Kit, One-color (Agilent Technologies, Santa Clara, CA, USA). The labeled cRNAs were placed on an Arabidopsis (V4) Gene Expression Microarray, 4 × 44K (Agilent Technologies), hybridized and washed according to the manufacturer's instructions. The arrays were analyzed using an Agilent scanner with associated software. Raw signal intensities were extracted with Feature Extraction v10.10.1.1 (Agilent Technologies) and normalized per chip to the 75th percentile and per probe to medians. Feature extraction flag criteria allowed data filtering, and the probe signal was considered as well above background if 'detected' was selected for all replicates of at least one experimental condition to pass the filtering. Thus, we identified the genes expressed in the mesophyll protoplasts regenerated for 0 h, 6 h, 12 h and 24 h.

**Table S2.** Composition (μg/mg dry weight) of the supernatant collected after every extraction step of the cell wall preparation.

| | CDTA | $Na_2CO_3$ | 1M KOH | 4M KOH | Cellulose |
|---|---|---|---|---|---|
| Native | 39.72 | 80.42 | 103.22 | 74.52 | 432.38 |
| 3 h | 4.77 | 2.48 | 12.37 | 7.84 | 192.87 |
| 1 h | 4.41 | 1.59 | 9.16 | 7.23 | 133.18 |

**Cell Wall Fractionation by Sequential Extraction**

The protoplasts and suspension-cultured cells were homogenized in 100 mM $K_2HPO_4$ containing 1 M KCl 3% sucrose using Polytron-Aggregate (Kinematica, Luzern, Switzerland). After washing three times with the same buffer, the pellets were further washed with 80% ethanol, 80% acetone, methanol:chloroform mixture (1:1) and 100% ethanol. The pellets were treated with $\alpha$-amylase, washed with water and then were lyophilized (Labconco FZ-4.5, Asahi Life Science, Saitama, Japan). Pectic fractions were prepared by extraction with 50 mM CDTA and 50 mM $Na_2CO_3$. Hemicelluloses were successively extracted with 1 M KOH, followed by extraction with 4 M KOH, designated as the 1 M KOH fraction and 4 M KOH fraction, respectively. The cellulose fraction was extracted successively with 1 M acetic acid and 1 M sodium acetate (pH 5.0).

**Table S3.** Proportion of monosaccharides in the pectin (CDTA) fraction.

| | Fuc | Rha | Ara | Glc | Man | Xyl | GalA | GlcA |
|---|---|---|---|---|---|---|---|---|
| Native | 2.4 | 3.4 | 12.1 | 11.0 | 0.0 | 31.9 | 36.2 | 3.0 |
| 3 h | 0.9 | 1.3 | 2.9 | 32.9 | 8.3 | 36.8 | 14.4 | 2.6 |
| 1 h | 0.6 | 0.4 | 1.2 | 41.0 | 8.2 | 46.4 | 1.8 | 0.5 |

Data are expressed as the percentage of all monosaccharides detected in the fraction.

**Analysis of Glycosyl Compositions of the Pectin Fraction**

CDTA (trans-1,2-diaminocyclohexane-*N,N,N',N'*-tetraacetic acid) fractions were hydrolyzed with 2 M trifluoroacetic acid (TFA) for 1 h at 121 °C, followed by evaporation under a reduced pressure using Speed Vac (SC110A, Savant Instrument, Inc., Farmingdale, NY, USA) to complete removal of TFA. The monosaccharides were re-dissolved in water and separated by HPAEC-PAD on a Dionex DX-500 (Dionex, Sunnyvale, CA, USA), which used a concentration gradient of 0.7 M sodium acetate and 0.2 M NaOH. Fuc; fucose, Rha; rhamnose, Ara; arabinose, Glc; glucose, Man; mannose, Xyl; xylose, GalA; galacturonic acid, GlcA; glucuronic acid.

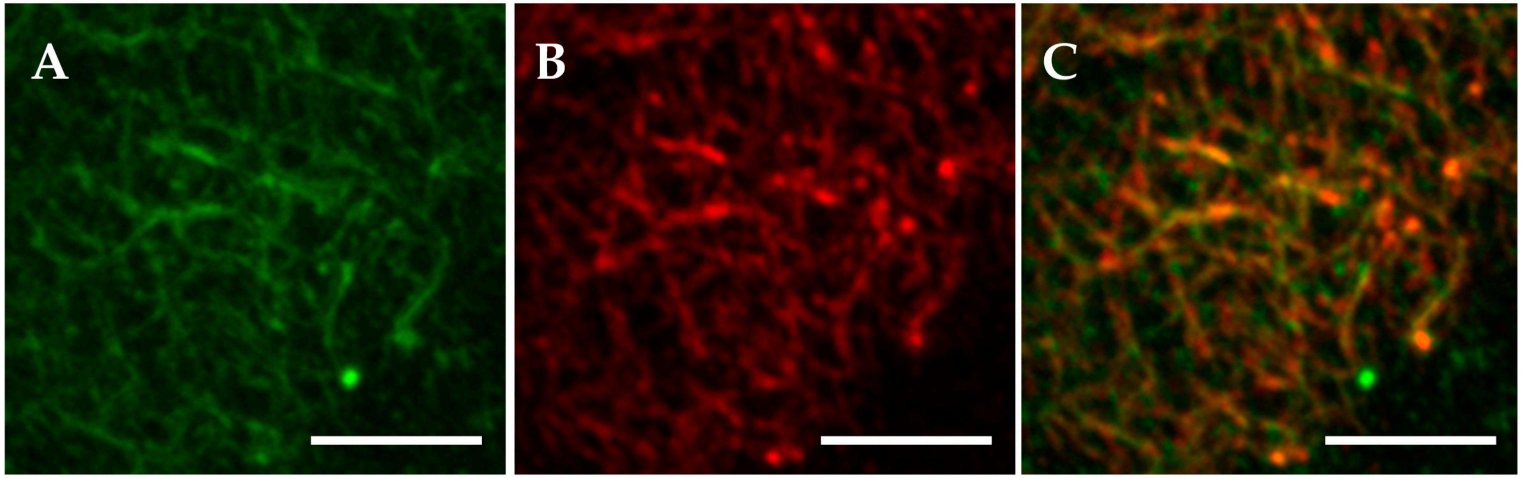


**Figure S1.** Localization of xyloglucan molecules and β-glucan on plasma membrane sheets of the regenerating protoplasts**.** (**A**) Staining of β-glycans with Calcofluor White; (**B**) immunofluorescence detection of LM15 binding to xyloglucan; (**C**) composite images of double labeling with Calcofluor White and LM15. Bar = 5 μm. Indirect immunofluorescence analysis was conducted according to a previously described method (https://www.plantcellwall.jp/protocol/pdf/protocol_8.pdf [1].

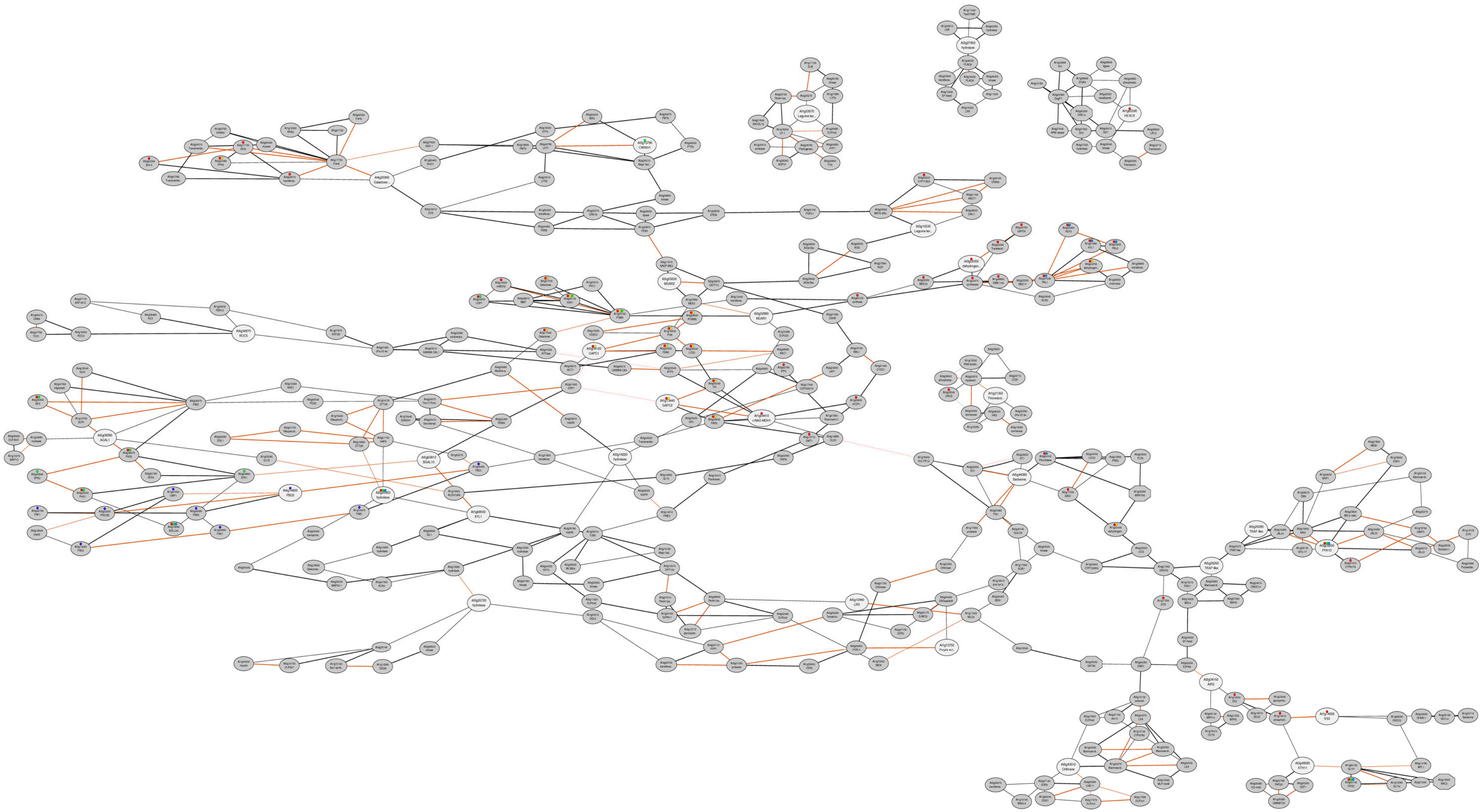

**Figure S2.** The co-expression network of the genes identified in the regenerating protoplasts. We focused on the genes encoding the proteins with putative secretory signal peptide, which were identified in the regenerating protoplasts, and filtered them through the ATTEDII co-expression database. Circles with white background indicate the genes identified in the regenerating protoplasts.

## Reference

1. The Plant Cell Wall Biology Laboratory, Tohoku University (2013) Indirect immunofluorescence assay with cell wall polysaccharide-directed monoclonal antibody for plant cells and tissues. https://www.plantcellwall.jp/protocol/pdf/protocol_8.pdf (accessed on 16 November 2016).
